# *Cryptosporidium parvum* infection alters the intestinal mucosa transcriptome in neonatal calves: impacts on epithelial barriers and transcellular transport systems

**DOI:** 10.3389/fcimb.2024.1495309

**Published:** 2024-12-04

**Authors:** Arash Veshkini, Christa Kühn, Franziska Dengler, Lisa Bachmann, Wendy Liermann, Christiane Helm, Reiner Ulrich, Cora Delling, Harald M. Hammon

**Affiliations:** ^1^ Research Institute for Farm Animal Biology (FBN), Dummerstorf, Germany; ^2^ Friedrich-Loeffler-Institute, Greifswald-Insel Riems, Germany; ^3^ Agricultural and Environmental Faculty, University Rostock, Rostock, Germany; ^4^ Institute of Animal Sciences, University of Hohenheim, Hohenheim, Germany; ^5^ Faculty of Agriculture and Food Science, University of Applied Science Neubrandenburg, Neubrandenburg, Germany; ^6^ Institute for Veterinary Pathology, Leipzig University, Leipzig, Germany; ^7^ Institute of Veterinary Parasitology, Leipzig University, Leipzig, Germany

**Keywords:** cryptosporidiosis, intestinal permeability, epithelial barriers, SLC transporter, ABC transporter, bovine

## Abstract

**Introduction:**

*Cryptosporidium parvum (C. parvum)* is the most prevalent enteric protozoan parasite causing infectious diarrhea in neonatal calves worldwide with a direct negative impact on their health and welfare. This study utilized next-generation sequencing (NGS) to deepen our understanding of intestinal epithelial barriers and transport mechanisms in the pathophysiology of infectious diarrhea in neonatal calves, which could potentially unveil novel solutions for treatment.

**Methods:**

At day 1 of life, male Holstein-Friesian calves were either orally infected (n = 5) or not (control group, n = 5) with *C. parvum* oocysts (in-house strain LE-01-Cp-15). On day 8 after infection, calves were slaughtered and jejunum mucosa samples were taken. The RNA was extracted from collected samples and subjected to sequencing. Differentially expressed genes (DEG) between the infected and CTRL groups were assessed using DESeq2 at a false discovery rate < 0.05 and used for gene ontology (GO) and pathway enrichment analysis in Cytoscape (v3.9.1).

**Results and discussion:**

To study the pathophysiology of infectious diarrhea on intestinal permeability, 459 genes related to epithelial cell barrier integrity and paracellular and transmembrane transport systems were selected from 12,908 identified genes in mucus. Among, there were 61 increased and 109 decreased gene transcripts belonged to adhesion molecules (e.g. ADGRD1 and VCAM1), ATP-binding cassette (ABC, e.g. ABCC2 and ABCD1) and solute carrier (SLC, e.g. SLC28A2 and SLC38A3) transporters, and ion channels (e.g. KCNJ15). Our results suggest deregulation of cellular junctions and thus a possibly increased intestinal permeability, whereas deregulation of ABC and SLC transporters and ion channels may influence the absorption/secretion of amino acids, carbohydrates, fats, and organic compounds, as well as acid-based balance and osmotic hemostasis. Besides pathogen-induced gene expression alterations, part of the DEG may have been triggered or consequently affected by inflammatory mechanisms. The study provided a deeper understanding of the pathophysiology of infectious diarrhea in neonatal calves and the host-pathogen interactions at the transcript level. For further studies with a particular focus on the transport system, these results could lead to a new approach to elucidating pathophysiological regulatory mechanisms.

## 1 Introduction

Neonatal calves (< 1-2 month old) are immunologically naïve and vulnerable to infectious diarrhea, a worldwide serious disease often caused by the protozoa *Cryptosporidium parvum* (*C. parvum*) with clinical symptoms including profuse watery stools, dehydration, acidosis, lethargy, and in rare cases, death (Lombardelli et al., 2019). *C. parvum* develops within a parasitophorous vacuole extracytoplasmatically but intracellularly in intestinal epithelial cells (IEC), primarily in the ileum, but also distal jejunum, and leads to profound changes in the IECs’ morphology, physiology, and transcription. *C. parvum* intestinal infection results in partial enlargement and swelling of lymph nodes, mild to moderate villus atrophy, detrimental effects on the mucosal and epithelial integrity, hindering normal absorption and secretion mechanisms, impairing the epithelial barrier function, and leading to an increase in intestinal permeability and leaky gut syndrome (Dumaine et al., 2021; Helmy and Hafez, 2022; Gamsjäger et al., 2023).

The intestinal barrier (IB) is primarily composed of the epithelium tightly sealed by tight junctions (TJ) and a mucus layer on top, which regulates nutrient absorption and functions as a physical barrier against harmful pathogens (Rios-Arce et al., 2017). The TJ are composed of several transmembrane proteins, including occludin (OCLN), claudins (CLDN), and immunoglobulin superfamily proteins, including junctional adhesion molecules (JAM), which interact with cytoskeletal linker proteins such as zonula occludens (ZO), forming a complex architecture that activates a multitude of cellular processes to maintain barrier integrity (Ahn et al., 2016; Usuda et al., 2021). There are other molecules and junctions located below TJ, such as adherens junctions (AJ), desmosomes, gap junctions (GJ), cell adhesion molecules (CAMs), and G protein-coupled receptors (GPCRs) superfamily, that are involved in cell-cell adhesion or binding to extracellular matrix (Schnell et al., 2013). Pathogens have evolved a variety of tactics to exploit junctional structures or destroy them and thereby providing a pathway into the underlying tissue, often provoking inflammatory cascades and diarrhea (Guttman and Finlay, 2009). A pathogen’s effects on intestinal permeability not only increase the risk of other pathogens’ invasion, but also influence absorption mechanisms, nutrient pools, and systemic metabolism (Aurora and Sanford, 2015; Di Vincenzo et al., 2024).

The major pathway for vectorial nutrient absorption across IECs is transcellular transport, which involves specific transporter proteins such as the ATP-binding cassette (ABC) transporter and the solute carriers (SLC) superfamilies (Klaassen and Aleksunes, 2010). In active transport, ABC transporters export substrates from cells in an energy-dependent manner, whereas in secondary active transport, members of the SLC family transport substances from the extracellular space (influx facilitated transporters) through electrochemical potential differences or ion gradients generated by primary active transporters (Haberkorn et al., 2021). SLC and ABC transporters can transport a wide variety of molecules, ranging from simple ions, sugars and amino acids to complex substrates such as lipids, vitamins, proteins, and xenobiotics, with a broad range of specificity from low to high even within a family. Transporters are essential for maintaining metabolic homeostasis, but also play a key physiological role in many cellular functions. Thus, deficits in transporters, even in one, depending on their degree of specification, can cause serious health problems (Lin et al., 2015). In addition to transporters, channels are also important players in the transport system, facilitating transport of water and other small solutes across biological membranes (Hodges and Gill, 2010). However, this area has been largely overlooked in the pathophysiology of infectious diarrhea in neonatal calves.

The recent development of high-throughput RNA-Sequencing (RNA-Seq) technologies has provided hypothesis-neutral information about the composition and abundance of transcriptomes. Combined with bioinformatics analysis, this could provide unique insight into a pathophysiological condition that is usually hard to model. Developing management and treatment for *cryptosporidiosis* can be greatly influenced by a deep understanding of the epithelial barriers, junctions, and transport mechanisms. The objective of the present study was therefore to investigate different aspects of intestinal cell junctions as well as substrate translocation in neonatal calves infected with *C. parvum* using next-generation sequencing (NGS).

## 2 Material and methods

### 2.1 Experimental design and sample collection

This study was framed under a recent comprehensive project described in detail by (Dengler et al., 2023) in accordance with the German legislation on the protection of animals, and licensed by the Landesdirektion Leipzig as TVV 19/20. In brief, ten healthy male neonatal calves (*Bos taurus*, Holstein-Friesian) were selected and transported to the University of Leipzig within their first 24 h of life. At day 1 of life, calves were infected by oral application of 2 × 10^7^ *C. parvum* oocysts (infected, n=5) or received pure water (CTRL, n = 5). Infection was confirmed through regular clinical monitoring of calves, measurement and scoring of fecal consistency, and examination of fecal shedding of *C. parvum* oocysts using the immunofluorescence assay kit MERIFLUOR^®^ Cryptosporidium/Giardia (Meridian Bioscience, Inc., Cincinnati, USA). In addition, a snap test (BoDia, Fassisi, Göttingen, Germany) was used to test the presence of E. coli K99, rotavirus, coronavirus, and *C. parvum* in fecal samples before and 7 days post infection. After slaughtering the calves on day 7 post infection, the jejunum epithelium was manually stripped off from the underlying muscle and stored at -80°C.

### 2.2 RNA extraction, quantification, library construction, and sequencing

The RNA extraction, library preparation, and NGS analysis was previously described in detail (Veshkini et al., 2024). In brief, individually extracted RNA (NucleoSpin RNA kit, Macherey-Nagel, Düren, Germany) from each sample was assessed for RNA integrity number and quantified using a Bioanalyzer (Agilent Genomics, Waldbronn, Germany) and Qbit (Fisher Scientific), respectively. mRNA transcriptome libraries were synthesized using Illumina TruSeq Stranded mRNA library preparation kits (Illumina, San Diego, USA) and 1 microgram of total RNA as an input, and sequenced on an HiSeq2500 (Illumina, San Diego, USA) with 2 x 100 bp cycles.

### 2.3 Data processing, read mapping, and statistical analysis

After demultiplexing, the obtained reads were analyzed for quality control, trimming, alignment and expression counts at gene and transcript level using the nf-core RNAseq v3.4 pipeline (https://nf-co.re/rnaseq/3.12.0) with default settings. For read alignment, the ARS-UCD1.2_Btau5.0.1Y assembly run 9 (Hayes and Daetwyler, 2019) without unplaced NKLS contigs served as backbone. The *Bos taurus* Ensembl genome annotation v105 provided gene and transcript coordinates. Gene expression counts were calculated via Salmon (v. 1.5.2) within the nf-core RNAseq pipeline.

Differentially expressed genes (DEG) were assessed by setting up an infection versus control group model in the DESeq2 1.26.0 package (Love et al., 2014) and limited to the transcripts having a transcript per million (TPM) value > 1 in at least 4 samples. Calculated P-values were adjusted for multiple testing via Benjamini-Hochberg. Only genes with a false discovery rate (FDR)_BH_ < 0.05 were accepted as statistically significant.

### 2.4 Statistical and bioinformatic analysis

The bioinformatic analysis was limited to a subset of DEG related to junctions (TJ, AJ, GJ), CAM, GPCRs, transporters, and ion channels (termed “barriers and transporters-DEG”, **Supplementary Table S1**) due to the large number of identified DEG. The immune-related DEG were discussed elsewhere (Veshkini et al., 2024). In order to select candidate genes, we searched our database against public databases such as STRING (Version 12), DAVID (https://davidbioinformatics.nih.gov/, version 2023 q1), and TransportDB (version 2.0). Gene ontology (GO) annotation and functional enrichment analysis including biological process and cellular component was executed by ClueGo (v2.5.9) in Cytoscape software according to the following criteria: FDR < 0.05 and having at least two identified DEG within each pathway (Bindea et al., 2009, 2013). The network nodes and edges data were retrieved from the ClueGo plugin and plotted using yFiles radial layout in Cytoscape software (v3.9.1).

## 3 Results

### 3.1 RNA sequencing

**Figure 1:**
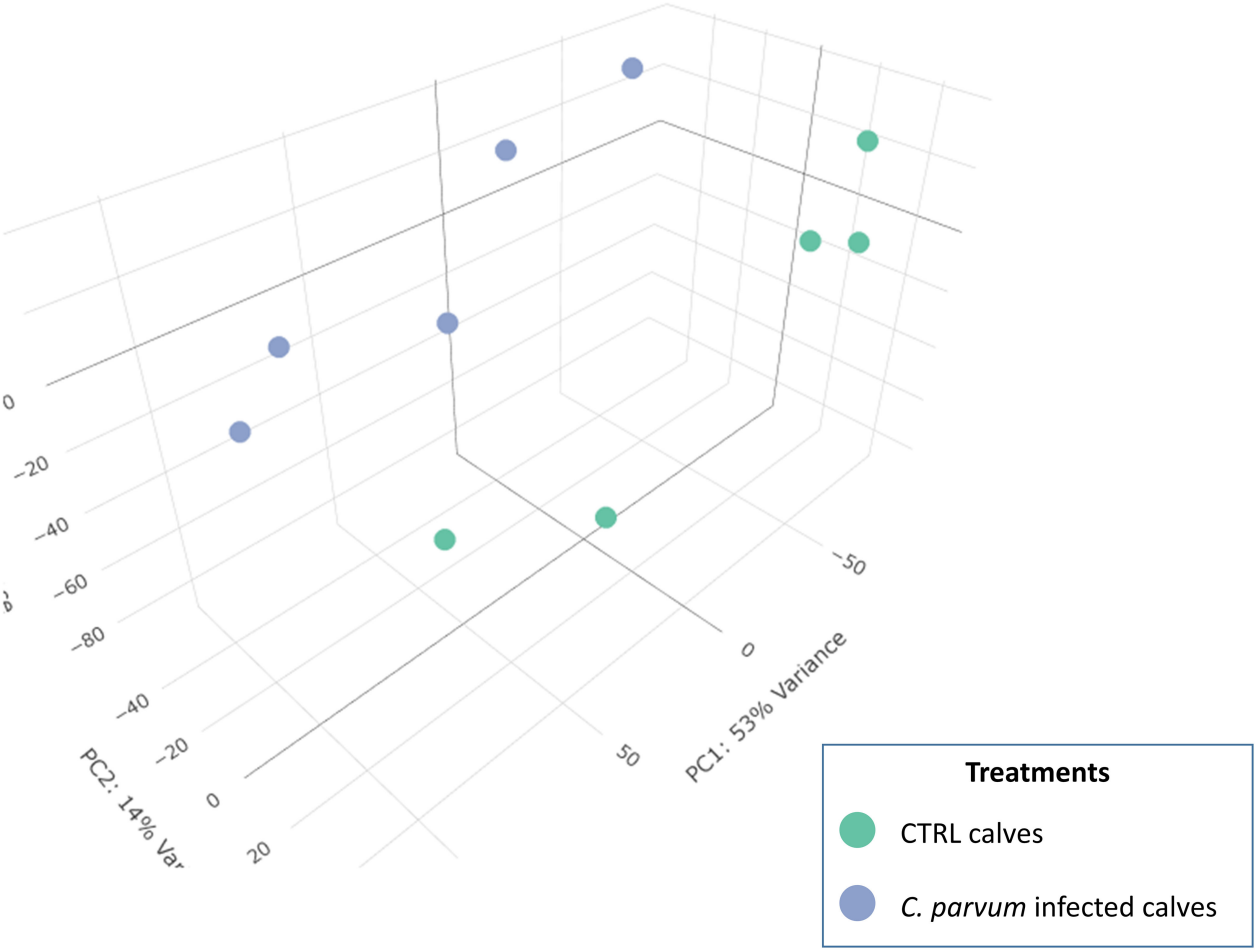
3D Principal component analysis (PCA) of the normalized RNAseq data in calves infected or not with *Cryptosporidium parvum*. A PCA is an unsupervised machine learning model that identifies individuals with similar characteristics to bring out strong patterns from large and complex datasets. The dots represent individual calves and are grouped into two recognizable clusters based on treatment groups (infected or not with *C. parvum*). This observation emphasizes that the treatment effect is the primary factor affecting the gene expression results in the current dataset. There is a value associated with each component (PC), which indicates its variance percentage explained by the model.

In the mucosal cells, 12,908 expressed genes at TPM >1 in at least four samples were identified, including 11,844 known genes and 1,064 genes with yet unknown function (ENSBTAG) (**Supplementary Table S2**). Principal component analysis (PCA) was used to compare gene expression patterns in mucosal cells collected from infected and non-infected calves (**Figure 1**). Despite unsupervised analysis, the two groups stand as two well-separated clusters, which indicates large differences in gene expression between the two groups related to the infection. The PC1 and PC2 together explain nearly 70% of the total variation.

### 3.2 Differentially expressed gene analysis between infected and non-infected calves and their associated biological pathways

**Figure 2:**
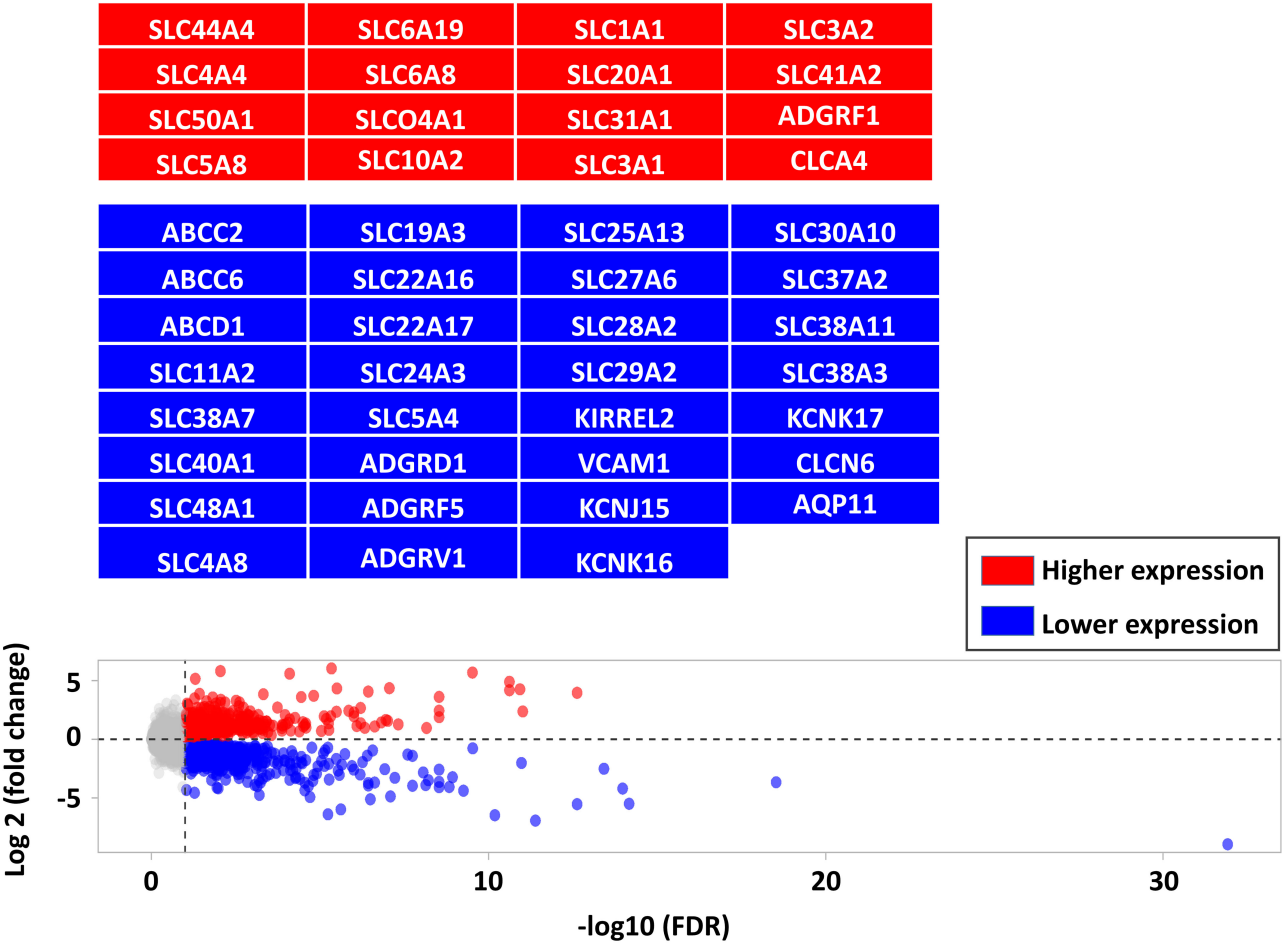
Comparing the differentially expressed genes (DEG, FDR < 0.05) between CTRL and infected calves. DEG are shown in red (higher expressed in infected calves) and blue (lower expressed in infected calves) and highlighted in corresponding boxes.

There were 562 lower and 405 higher expressed genes at a FDR < 0.05, log2-fold change range: ≈ -6 to +6 (**Supplementary Table S3**). As a complement to our latest study focused on immune markers, this study focused on barriers and transporters-DEG including transcripts of 460 gene encoding TJ, AJ, GJ, CAM, ATPase transporters, ABC transporters, SLC transporters, and channels (**Supplementary Table S1**). Among barrier- and transporter-DEGs, there were 59 genes with higher expression and 109 genes with lower expression in infected calves as highlighted in the **Figure 2** (the full list is provided in **Supplementary Table S4**).

**Figure 3:**
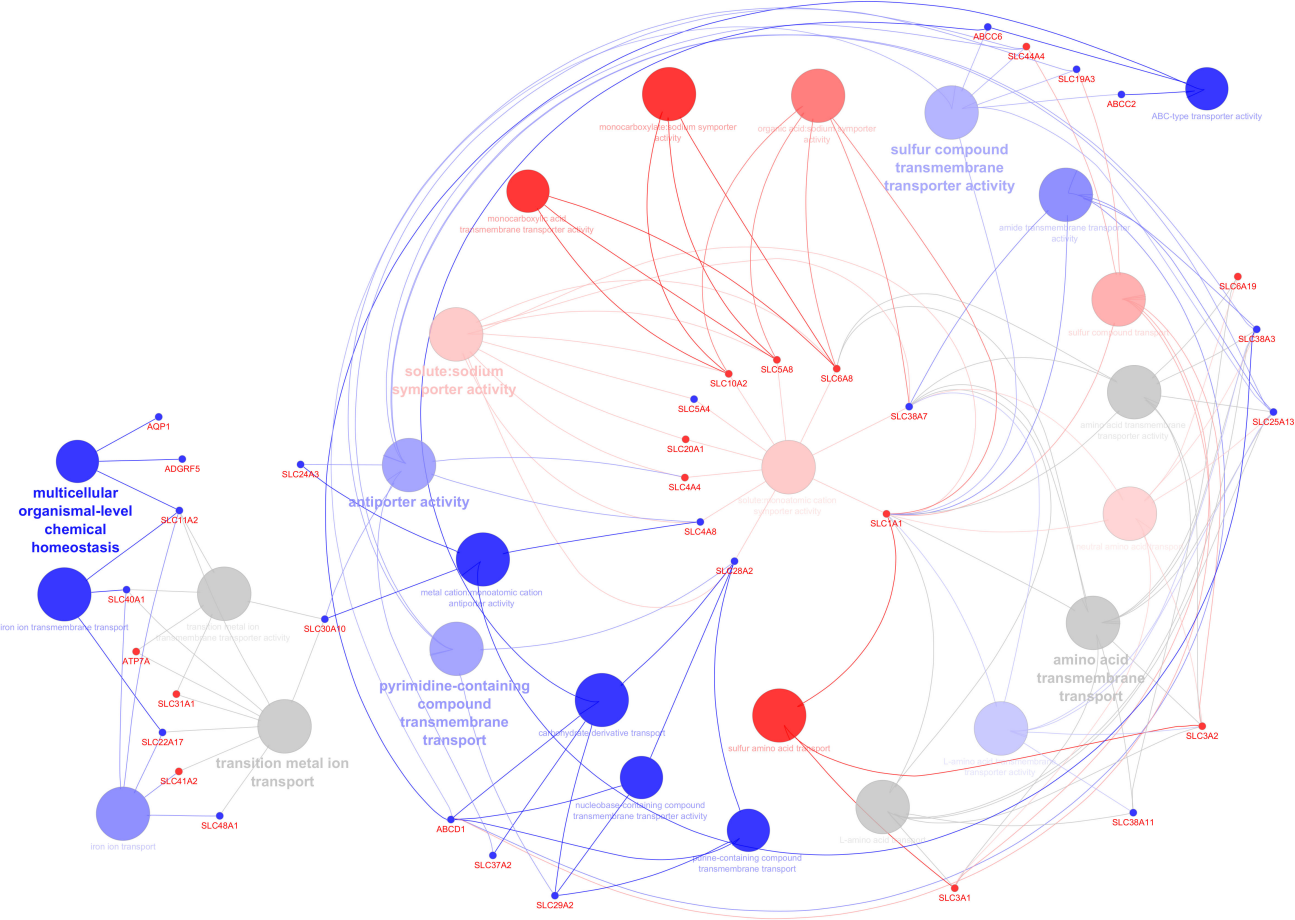
Predicting the biological processes’ direction associated with differentially expressed genes in the infected calves. The network shows interconnections among different biological pathways. Biological pathways are colored according to their predicted upregulation (red), downregulation (blue), or neutral (gray, the direction is not distinguishable). The hue intensity corresponds to false discovery rate. The highlighted genes are those that were initially or centrally involved in pathway enrichment, with red and blue dots indicating genes with higher and lower expression, respectively.

*C. parvum* infection significantly impacted the transcript expression of AJ, CAM, GPCR, transporters, and channels but not TJ or GJ. Gene ontology analysis was performed to predict up- and downregulated genes’ biological processes and their directions in a holistic manner and highlighted the key genes involved in the network (**Figure 3**). Among the pathways annotated to the DEG are those involved in the transport and metabolism of organic acids, carbohydrate derivate, lipids and fatty acids, amino acids, and ions (cations and anions) (**Supplementary Table S5**). The network shows deregulated pathways involved in transmembrane transport of ions, carbohydrates, and other organic compounds, affecting the cellular homeostasis.

**Figure 4:**
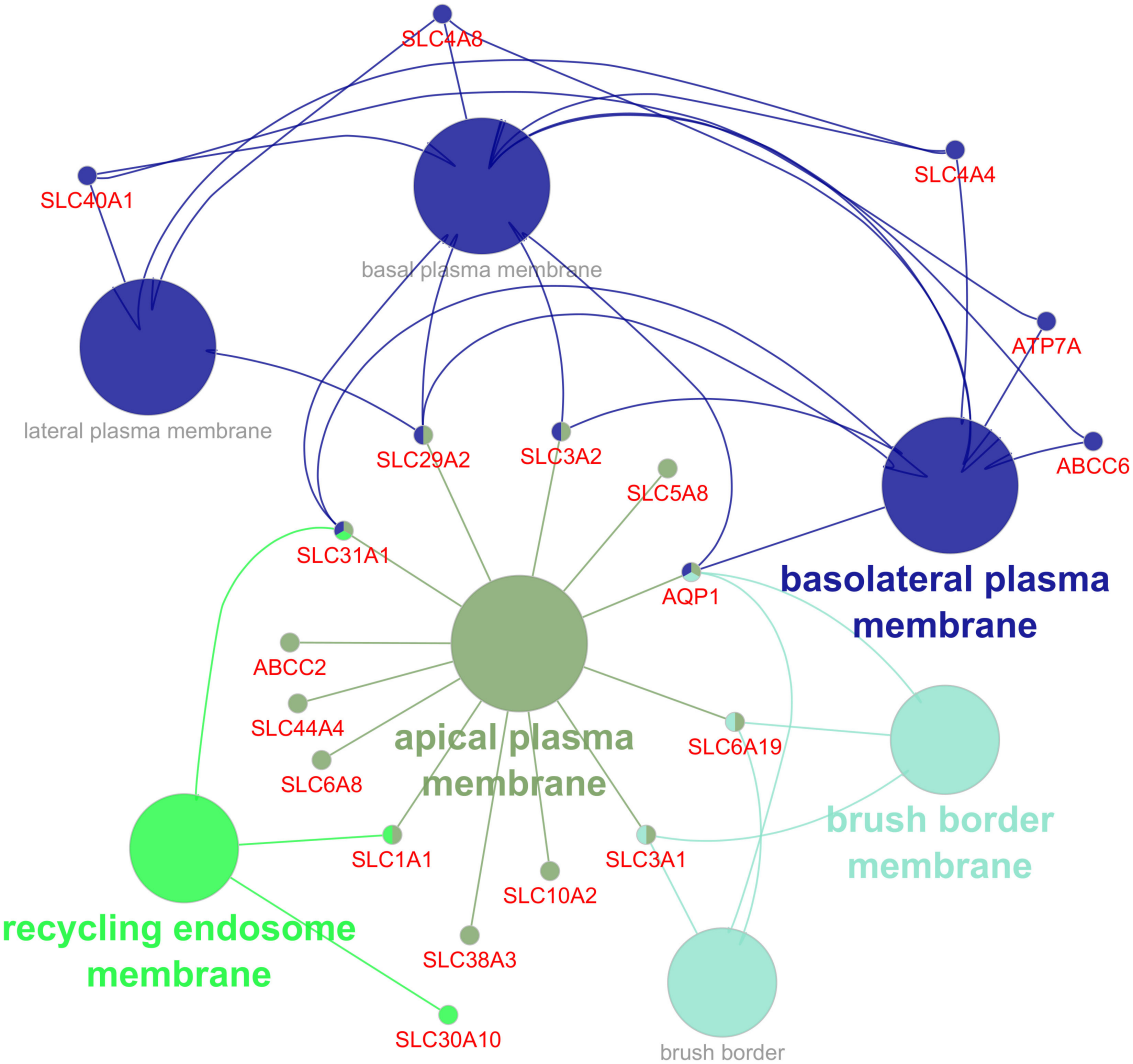
Gene ontology (GO) functional enrichment analysis of cellular component (CC) annotated to differentially expressed genes in the infected group. Different colors indicate clusters automatically assigned by the ClueGo, with circles representing pathways and dots representing genes.

At cellular component level, DEGs were annotated to various cell compartments including the morphological structure of the cellular membrane such as basolateral, and brush border as well as intracellular organelles such as endosome and endoplasmic reticulum (**Figure 4**; **Supplementary Table S6**). The number of differentially expressed cell membrane-associated genes are higher in the apical part of the cell than in the basolateral part.

## 4 Discussion

There has been evidence that *C. parvum* infection is associated with the disruption of the host’s intestinal epithelial barrier integrity and increased permeability; however, the underlying molecular mechanisms in dairy calves have not been completely elucidated. We have previously reported the impact of *C. parvum* on calves’ clinical health status, zootechnical performance, and immunohematological parameters (Dengler et al., 2023; Veshkini et al., 2024). In brief, our histopathological results showed that *C. parvum*-infected calves had villus atrophy and reduced villus-crypt ratios, suggesting tissue damage (**Supplementary Figure S7**). The possibility of cellular damage by *C. parvum* may also be supported by the enrichment of oxidative stress, apoptosis, and programmed cell death signaling pathways in the jejunum mucosa of the infected calves (**Supplementary Table S8**). Additionally, *C. parvum* infection was confirmed at the jejunum tissue by comparing the abundance of oxidative stress, apoptosis, and programmed cell death related proteins extracted from our proteomics analysis (**Supplementary Figures S9, S10**).

The infected calves had lower glucose and higher urea plasma concentrations, but body weight, internal body temperature, and immunohematology markers were not significantly changed. Regarding the immune system, we have found that inflammatory signaling pathways such as Toll-like receptors (TLRs) and the nuclear factor-kappa B (NF-κB) are induced following infection, but the adaptive immune response is not yet fully responsive (Veshkini et al., 2024). This study complements those findings and mainly focused on aspects of calf intestinal nutrient transport during *C. parvum* infection. There are distinct pathways used by the intestinal epithelium to transport substances from the gut lumen into the circulation or vice versa, depending on their size, hydrophobicity, and physicochemical characteristics. To better comprehend the effect of *C. parvum* on intestinal permeability and pathophysiology of diarrhea, the following sections will discuss the molecular aspects of each pathway separately.

### 4.1 Paracellular transport and intestinal permeability regulation

Paracellular transport involves passive but selective transport of substances across an epithelium through cell junctions, including TJ, AJ, GJ, desmosomes, and hemidesmosomes, which are arranged into at least two distinct pathways: high-capacity pores (mainly regulated by CLDN family) and low-capacity leaks (regulated by OCLN and ZO family) (Zheng et al., 2021). During stimulation or pathological conditions, disruption of these junctions’ integrity can increase paracellular permeability, allowing pathogens to enter the systemic circulation and affect systemic immune function (Chelakkot et al., 2018).

*C. parvum* infection influenced the expression of calcium-dependent adhesion (cadherins superfamily) through reduction of cadherin related family member 1 (CDHR1) and cadherin 13 (CDH13), and induction of CDH26 and protocadherin 1 (PCDH1). The cadherin family is required for the formation of AJ (George and Beeching, 2006) and consequently TJ (McCole, 2014), thus their deregulation may reflect on barrier functions. In accordance, studies in mice and humans have shown that *C. parvum* infection downregulates components of TJs and AJs to translocate through the intestinal mucosa and invade their host (Kumar et al., 2018; Lamisere et al., 2022). There is evidence that the physical damage to the epithelial cells by *C. parvum* contributes to increased intestinal permeability or leaky gut syndrome in calf and mice models (De Sablet et al., 2016; Gamsjäger et al., 2023), in which dysregulation of AJs might be one of the reasons. In contrast to TJs, which seal paracellular gaps between adjacent epithelial cells and maintain electrochemical gradients necessary for efficient transcellular ion transport (Tsukita et al., 2001), AJs provide mechanical linkages (cell–cell contacts) between adjacent epithelial cells (Yap et al., 1997). This evidence suggests that *C. parvum* infection may impair junctions integrity, resulting in increased intestinal permeability, and in turn facilitating secondary infections.

Other adhesion molecules, such as those belonging to the CAM and GPCR families, were also affected by infection, including a lower expression of vascular cell adhesion molecules 1 (VCAM1), Kirre-like nephrin family adhesion molecule 2 (KIRREL2), adhesion G protein-coupled receptors (ADGR)-D1, ADGRF5, and ADGRV1, and a higher expression of ADGRF1. Unfortunately, the role of CAM and GPCR families in the pathophysiology of diarrhea in ruminants is poorly understood. Gut permeability and development also depend heavily on inflammatory and immune responses (Jridi et al., 2020). As a part of interactions between hosts and pathogens, tumor necrosis factor (TNF)-α and interleukin (IL)-1β produced by inflammatory monocytes aid *C. parvum* in changing the intestinal barrier and permeability (De Sablet et al., 2016). A previous mouse model experiment revealed that *C. parvum* infection altered immunohistochemical localization of Wnt pathway components as well as adherens junction ultrastructure in ileocecal epithelium (Benamrouz et al., 2014). TNFα signaling induces VCAM-1 expression, which regulates inflammation-related vascular adhesion and leukocyte migration across the endothelial barrier and entry into sites of immune complex deposition (Kong et al., 2018). Also, the ADGR family appears to transmit external signals, such as stimulants, to induce physiological responses that regulate mucosal immunity and maintain intestinal barrier function (Sun et al., 2017). At one side, physical damage caused by *C. parvum* infection affects TJ and AJ’s regulation and localization; however, inflammation and immune responses may also be their regulators.

### 4.2 Transmembrane transport

#### 4.2.1 ATP-binding cassette and ATPases transporters

The primary active transporters are ATP-dependent, including the family of ABC transporters and ATPases (ion pumps) that transport ions, lipids, carbohydrates, and xenobiotics against a concentration gradient (Schumann et al., 2020). ABC transporters play a crucial role in the pathophysiology of infectious diarrhea for two reasons: they facilitate secretion of various ions such as chloride and modulate xenobiotic absorption, distribution, metabolism, and secretion, but they can also interact with pathogens, potentially contributing to host protection (Mercado-Lubo and McCormick, 2010).

A number of ABC transporter genes were expressed in our data set (**Supplementary Table 2**), but only ABCC2, ABCC6, and ABCD1 expressions were reduced by infection. Nies and Keppler (2007) previously reviewed the substrate specificity of human and rat ABCC2. In the gut epithelium, ABCC2 localizes exclusively in the apical brush border membrane of villi, serving to efflux anions such as chloride and excrete endogenous and xenobiotic substances, especially in conjunction with glutathione, glucuronate, or sulfate into the intestinal lumen (Nies and Keppler, 2007). During intestinal infection (by Salmonella typhimurium), ABCC2 was shown to be upregulated (Pazos et al., 2008), probably to facilitate the apical efflux of the neutrophil chemoattractant hepoxilin A3 (HXA3) (Mrsny et al., 2004). Even though *C. parvum* induces intestinal inflammation, we have previously shown that neutrophil chemotaxis and differentiation associated pathways were downregulated (Veshkini et al., 2024), suggesting a minor role of neutrophils in neonatal calves right after the peak of infection. ABC transporter expression is under the control of transcription factors such as the retinoid X receptor (RXR), pleomorphic adenoma gene (PLAG), and zinc finger transcription factor specificity protein 1 (SP1) which imprints a tissue-specific expression (Abruzzese et al., 2022). In contrast to ABCC2, the ABCC6 transporter is located at the basolateral membrane and serves multiple functions, however the precise nature of its substrate(s) is unknown (Vanakker et al., 2013). It can be postulated that nutrient drainage by *C. parvum* interferes with the ABCC2 and ABCC6 function and expression, thereby disrupting the secretion and homeostasis of one or more (yet unknown) substrates. Furthermore, ABCD1, which is located at the peroxisome membrane, facilitates the import of coenzyme A-activated saturated very long-chain fatty acids (VLCFA; >C22:0) into peroxisomes for break down by β-oxidation, a crucial step in activating innate immunity, especially pro-inflammatory signaling in monocytes and macrophages (Zierfuss et al., 2022). Since fatty acids are required for both glycolysis and lipogenesis to cover the rapid energy demands and produce pro-inflammatory mediators, a downregulation of ABCD1 appears to interfere with the immune response and lead to the accumulation of VLCFA in plasma and tissues.

In addition, *C. parvum* infection affected members of ATPase transporters, resulting in higher expression of ATPase Ca^2+^ transporting 2 (ATP2A2) and ATPase Copper Transporting Alpha (ATP7A). ATP2A2 is an intracellular pump primarily located in the sarcoplasmic reticula and endoplasmic reticula of muscle tissues (Dhitavat et al., 2003). It functions as a regulator of the contraction/relaxation cycle by hydrolyzing ATP and translocating calcium from the cytosol into the sarcoplasmic reticulum lumen, thus probably playing a role in the intestinal motility to compensate diminished absorption. This claim is, however, unsupported by any evidence, and further research is required to approve it.

#### 4.2.2 SLC transporters

Solute carrier proteins (SLC) are a large and diverse group of membrane proteins which mediate passive and secondary active transport of ions, nucleotides, and sugars across biological membranes. In this study 240 solute carrier proteins (SLC) were identified in our data set (**Supplementary Table 2**), of which 18 genes had higher and 14 genes had lower expression levels (**Figure 2**). Bioinformatics analysis suggested that the transport and homeostasis of cation, anion, and metal ion, carboxylic acid, sulfur, pyrimidine containing compounds, nucleobase containing compounds, purine containing compounds, and carbohydrate derivate were disturbed due to the lower expression levels of SLC48A1, SLC22A17, SLC40A1, SLC11A2, SLC30A10, SLC37A2, SLC29A2, SLC24A3, SLC4A8, SLC5A4, SLC19A3 and the higher expression levels of SLC3A2, SLC20A1, SLC6A19, SLC31A1, SLC4A4, SLC5A8, SLC10A2, SLC6A8 (**Figure 3**; **Supplementary Table S5**).

In the pathophysiology of diarrhea, dysregulation of intestinal ion transport is a critical factor (Das et al., 2018). Some of the differentially expressed SLC transporters are involved in the ion transport system across (in or out) cell membranes. In particular, influx divalent metal transporter 1 (DMT1), also known as SLC11A2, is the solute transporter responsible for transport of ferrous iron across the brush border membrane of intestinal epithelial cells (Kayaaltı et al., 2015). Afterwards, the basolateral efflux channel ferroportin (FPN, SLC40A1) mediates the intracellular translocation and export of iron into the circulation in conjunction with multicopper ferroxidase, including hephaestin (Heph) and/or ceruloplasmin (Cp), and hepcidin (Dlouhy et al., 2019). These two SLC transporters are exclusively iron transporters, and their downregulation in our study indicates diminished iron turnover. Iron availability is crucial not only to support erythropoiesis, immune cell differentiation and function, metabolic processes, and cellular respiration but also essential for pathogens’ proliferation and pathogenicity (Grander et al., 2022). There is evidence indicating a close relationship between DMT1 and FPN transporters and inflammation. Previous work reported that intestinal inflammation interferes with iron metabolism and DMT1 expression, affecting erythropoietic (Urrutia et al., 2013) and macrophage function (Grander et al., 2022). Activated Toll-like receptors (TLRs) and inflammatory factors such as IL-6 are known to induce the production of hepcidin, which consequently reduces iron availability by suppressing the expression of FPN1 through the hepcidin-FPN1 axis (Cai et al., 2021). TNF exposure reduced DMT1 expression in human intestinal cells (*in vitro*) (Johnson et al., 2004) and reduced duodenal iron transport in mice (*in vivo*) (Laftah et al., 2006). In accordance, we reported earlier that TNF-α is induced in response to *C. parvum* infection but neither erythrocytes and immune cell count nor hemoglobin and hematocrit concentrations were significantly affected (Veshkini et al., 2024).

The other group of differentially expressed SLC transporters in our study are involved into transport of more complex compounds such as sugars, amino acids, vitamins, nucleotides, carbohydrates, oligopeptides, and drugs. In the small intestine, thiamine (Vitamin B1) is transported across cell membranes by SLC19A3, where it is phosphorylated to its active form, thiamine pyrophosphate (TPP), and acts as a cofactor of transketolase. TPP is later transported by SLC25A19 to mitochondria, where it is a cofactor of pyruvate dehydrogenase complexes, branched chain ketothiolase dehydrogenases, and alpha-ketoglutarate dehydrogenases (Plecko and Steinfeld, 2017). SLC19A3 downregulation while SLC25A19 remains unchanged may affect the pyruvate oxidation in TCA cycle as well as energy and amino acids metabolism. SLC25A13, encoding a citrin protein, is an important mitochondrial solute transporter involved in the urea metabolism by swapping mitochondrial aspartate for cytosolic glutamate, where it can be used later for nucleotide synthesis pathways (Lin et al., 2011). Thus, it can be postulated that downregulation of SLC25A13 may affect the malate-aspartate shuttle, gluconeogenesis, amino acid homeostasis, TCA cycle, and protein turnover.

The higher expression levels of SLC transporters appears to be due to feedback mechanisms initiated by inflammation as compensation for vital compounds and also to regulate the activity of immune cells, but less information is available in cattle. ASBT is an active sodium-dependent bile acid transporter (encoded by SLC10A2) responsible for ileal bile acid (BA) reabsorption and has also been linked to intestinal disorders like diarrhea (Jung et al., 2004). Moreover, it plays an important role in regulating lipid and cholesterol homeostasis as well as determining the size of the BAs pool (Han et al., 2015). ASBT gene expression is primarily controlled by transcription factors such as caudal-type homeobox-1 (CDX1) and -2 (CDX2) and hepatocyte nuclear factor 1-α (HNF1-α), nuclear receptors such as retinoid X receptor (RXR) and farnesoid x receptor (FXR), and intestinal flora, which plays a critical role in both pathological and physiological processes as reviewed by (Yang et al., 2020). The reasons for the SLC10A2 higher expression is not clear but it seems to be a compensatory mechanism to reabsorb BA and decrease the excretion of fecal BAs and keep the liver cholesterol homeostasis in check.

Solute carrier family 1, member 1 (SLC1A1; also known as EAAT3 or EAAC1) is a major epithelial transporter of L-glutamate and D/L-aspartate especially in the intestines (Bailey et al., 2011; Sheng et al., 2022). The sodium bicarbonate cotransporter (NBCe1) encoded by SLC4A4 plays a role in pH regulation and homeostasis by transporting the bicarbonate from the blood to the lumen (Cappellesso et al., 2022). Positive correlations were found between SLC1A1 expression and levels of infiltrating CD8+T cells and dendritic cells, and between SLC4A4 expression and CD8+T cell infiltration levels (Zhou et al., 2020). A number of organic anion transporting polypeptides (OATPs) substrates are transported by SLCO4A1, including steroid hormone conjugates, pro-inflammatory prostaglandin PGE2, thyroid hormones, and xenobiotics. SLCO4A1 expression is under control of inflammation-associated pathways such as NF-κB, and TNF-receptor 2 signaling cascades (Koller et al., 2022). As immune cells depend on SLCs to induce rapid and robust metabolic reprogramming, thereby controlling their expression through major immune associated nuclear transcription factors, this seems to be a consequence of an activated immune response.

#### 4.2.3 Channels and membrane transporters with channel like properties

In our study, a subset of genes was identified to function as ion channels, notably chloride channels, potassium channels (KCN family), magnesium transporters, copper transporters, and heme transporters (**Supplementary Table 2**). Among those, the infected calves had lower expression of KCNJ15, KCNK16, and KCNK17, as well as chloride voltage-gated channel 6 (CLCA6), whereas calcium-sensitive chloride transporting channel 4 (CLCA4) had higher expression.

During infectious diarrhea, disruption of ion channel function may lead to alterations in electrolyte, nutrient, and fluid transport, potentially causing acid-base imbalances. In this regard, a good example would be the complex relationship between ion channels and lactose absorption in the intestinal epithelium under diarrheal conditions (Misselwitz et al., 2019). *C. parvum*-induced diarrhea leads to reduced lactase activity at the brush border, lower absorption of lactose metabolites and sodium via sodium/glucose cotransporter 1 (SGLT1), and increased passage of lactose to the distal small intestine and colon (Gomez et al., 2022). Due to the coupled transport of sodium and potassium with glucose and galactose, reduced lactose absorption means less glucose is available for SGLT1, potentially decreasing sodium absorption.

The function of potassium channels is closely connected with the (re)absorption of Na^+^, Cl^−^, and water as well as secretion of K^+^, HCO_3_^−^ in the basolateral and luminal membranes of the epithelial cells (Heitzmann and Warth, 2008). Chloride channels regulate intestinal Cl− secretion and have emerged as potential targets in the diarrhea treatment (Ko et al., 2014; Thiagarajah et al., 2015). At the crypt base, cells predominantly secrete fluid containing Cl^−^ and HCO_3_^–^ but the surface epithelial cells’ function is to reabsorb Na^+^ and to secrete HCO_3_^−^ and K^+^ to preserve the ion gradient at the crypt-villus axis (Heitzmann and Warth, 2008). Therefore, ion channel deregulation, leading to acid-base imbalances, as a part of physiologic and metabolic pathways leading to bacterial D-lactate production, could partly explain diarrheic calf metabolic (D-lactic) acidosis.

The absorption of chloride is essential for fluid absorption, and when dysregulated by infection, it affects the absorption of water and other solutes. In this regard, another cluster of DEG was associated with the transportation of solutes such as water and cholesterol (**Supplementary Table 4**). In response to infection, aquaporin (AQP11) expression was reduced, whereas ATPase transporters (ATP2A2, ATP7A) expression were significantly elevated. AQPs are water channel proteins that facilitate the absorption of fluid (primarily water) in many tissues including the small intestine and colon and are responsive to osmotic gradients (Zhu et al., 2016; Tomita et al., 2017; Zhang et al., 2019). The osmoregulation and mucosal fluid fluxes associated with ion and aquaporin channels make them a potential target for diarrhea treatment.

## 5 Conclusion

A thorough understanding of the pathophysiology of infectious diarrhea, including the absorption and excretion mechanisms, is the critical step for developing antidiarrheal agents. Using NGS and bioinformatics analysis, this study investigated how host-pathogen interactions may affect intestinal epithelial permeability and cellular transport mechanisms. The study highlighted critical DEGs encoding junctions, adhesions molecules, transporters, and channels associated with the pathophysiology of infectious diarrhea and provided an insight into their complex interactions, expressions, and regulations after infection. Among, infection-induced differential expression of adhesion molecules such as the cadherin family may affect intestinal permeability and paracellular absorption. Meanwhile, differential expressions of SLC and ABC transporters, along with ion and water channels, not only affect nutrient transmembrane transport, but also affect acid-base balance, electrochemical gradients, and osmotic homeostasis, which may partly contribute to metabolic acidosis in neonatal calves. Further studies and validation with additional methods should be conducted to determine which of these transporters of channels are potent candidate for designing new treatment strategies, whether these changes are related to pathogen impact or perhaps as a results of immune response preventing pathogens from obtaining host nutrients.

## Data Availability

The datasets presented in this study can be found in online repositories. The names of the repository/repositories and accession number(s) can be found in the article/**Supplementary Material**.

## References

[B1] Abruzzese V. Sukowati C. H. C. Tiribelli C. Matera I. Ostuni A. Bisaccia F. (2022). The expression level of ABCC6 transporter in colon cancer cells correlates with the activation of different intracellular signaling pathways. Pathophysiology 29, 173–186. doi: 10.3390/pathophysiology29020015 35645325 PMC9149812

[B2] Ahn C. Shin D. H. Lee D. Kang S. M. Seok J. H. Kang H. Y.. (2016). Expression of claudins, occludin, junction adhesion molecule A and zona occludens 1 in canine organs. Mol. Med. Rep. 14, 3697–3703. doi: 10.3892/mmr.2016.5725 27600198 PMC5042783

[B3] Aurora R. Sanford T. (2015). Host microbiota contributes to health and response to disease. Mo Med. 112, 317–322. 26455065 PMC6170062

[B4] Bailey C. G. Ryan R. M. Thoeng A. D. Ng C. King K. Vanslambrouck J. M.. (2011). Loss-of-function mutations in the glutamate transporter SLC1A1 cause human dicarboxylic aminoaciduria. J. Clin. Invest. 121, 446–453. doi: 10.1172/jci44474 21123949 PMC3007158

[B5] Benamrouz S. Conseil V. Chabé M. Praet M. Audebert C. Blervaque R.. (2014). Cryptosporidium parvum-induced ileo-caecal adenocarcinoma and Wnt signaling in a mouse model. Dis. Model. Mech. 7, 693–700. doi: 10.1242/dmm.013292 24652769 PMC4036476

[B6] Bindea G. Galon J. Mlecnik B. (2013). CluePedia Cytoscape plugin: pathway insights using integrated experimental and in silico data. Bioinformatics 29, 661–663. doi: 10.1093/bioinformatics/btt019 23325622 PMC3582273

[B7] Bindea G. Mlecnik B. Hackl H. Charoentong P. Tosolini M. Kirilovsky A.. (2009). ClueGO: a Cytoscape plug-in to decipher functionally grouped gene ontology and pathway annotation networks. Bioinformatics 25, 1091–1093. doi: 10.1093/bioinformatics/btp101 19237447 PMC2666812

[B8] Cai C. Zeng D. Gao Q. Ma L. Zeng B. Zhou Y.. (2021). Decreased ferroportin in hepatocytes promotes macrophages polarize towards an M2-like phenotype and liver fibrosis. Sci. Rep. 11, 13386. doi: 10.1038/s41598-021-92839-z 34183746 PMC8239022

[B9] Cappellesso F. Orban M.-P. Shirgaonkar N. Berardi E. Serneels J. Neveu M.-A.. (2022). Targeting the bicarbonate transporter SLC4A4 overcomes immunosuppression and immunotherapy resistance in pancreatic cancer. Nat. Cancer 3, 1464–1483. doi: 10.1038/s43018-022-00470-2 36522548 PMC9767871

[B10] Chelakkot C. Ghim J. Ryu S. H. (2018). Mechanisms regulating intestinal barrier integrity and its pathological implications. Exp. Mol. Med. 50, 1–9. doi: 10.1038/s12276-018-0126-x PMC6095905 30115904

[B11] Das S. Jayaratne R. Barrett K. E. (2018). The role of ion transporters in the pathophysiology of infectious diarrhea. Cell Mol. Gastroenterol. Hepatol. 6, 33–45. doi: 10.1016/j.jcmgh.2018.02.009 29928670 PMC6007821

[B12] Dengler F. Hammon H. M. Liermann W. Görs S. Bachmann L. Helm C.. (2023). Cryptosporidium parvum competes with the intestinal epithelial cells for glucose and impairs systemic glucose supply in neonatal calves. Veterinary Res. 54, 40. doi: 10.1186/s13567-023-01172-y PMC10156424 37138353

[B13] De Sablet T. Potiron L. Marquis M. Bussière F. I. Lacroix-Lamandé S. Laurent F. (2016). Cryptosporidium parvum increases intestinal permeability through interaction with epithelial cells and IL-1β and TNFα released by inflammatory monocytes. Cell Microbiol. 18, 1871–1880. doi: 10.1111/cmi.12632 27324279

[B14] Dhitavat J. Dode L. Leslie N. Sakuntabhai A. Lorette G. Hovnanian A. (2003). Mutations in the sarcoplasmic/endoplasmic reticulum ca2+ ATPase isoform cause darier’s disease. J. Invest. Dermatol. 121, 486–489. doi: 10.1046/j.1523-1747.2003.12410.x 12925205

[B15] Di Vincenzo F. Del Gaudio A. Petito V. Lopetuso L. R. Scaldaferri F. (2024). Gut microbiota, intestinal permeability, and systemic inflammation: a narrative review. Internal Emergency Med. 19, 275–293. doi: 10.1007/s11739-023-03374-w PMC10954893 37505311

[B16] Dlouhy A. C. Bailey D. K. Steimle B. L. Parker H. V. Kosman D. J. (2019). Fluorescence resonance energy transfer links membrane ferroportin, hephaestin but not ferroportin, amyloid precursor protein complex with iron efflux. J. Biol. Chem. 294, 4202–4214. doi: 10.1074/jbc.RA118.005142 30647129 PMC6422103

[B17] Dumaine J. E. Sateriale A. Gibson A. R. Reddy A. G. Gullicksrud J. A. Hunter E. N.. (2021). The enteric pathogen Cryptosporidium parvum exports proteins into the cytosol of the infected host cell. eLife 10, e70451. doi: 10.7554/eLife.70451 34866573 PMC8687662

[B18] Gamsjäger L. Cirone K. M. Schluessel S. Campsall M. Herik A. Lahiri P.. (2023). Host innate immune responses and microbiome profile of neonatal calves challenged with Cryptosporidium parvum and the effect of bovine colostrum supplementation. Front. Cell Infect. Microbiol. 13. doi: 10.3389/fcimb.2023.1165312 PMC10189047 37207189

[B19] George S. J. Beeching C. A. (2006). Cadherin:catenin complex: A novel regulator of vascular smooth muscle cell behaviour. Atherosclerosis 188, 1–11. doi: 10.1016/j.atherosclerosis.2005.12.017 16438974

[B20] Gomez D. E. Li L. Goetz H. Macnicol J. Gamsjaeger L. Renaud D. L. (2022). Calf diarrhea is associated with a shift from obligated to facultative anaerobes and expansion of lactate-producing bacteria. Front. Veterinary Sci. 9. doi: 10.3389/fvets.2022.846383 PMC8981386 35392114

[B21] Grander M. Hoffmann A. Seifert M. Demetz E. Grubwieser P. Pfeifhofer-Obermair C.. (2022). DMT1 protects macrophages from salmonella infection by controlling cellular iron turnover and lipocalin 2 expression. Int. J. Mol. Sci. 23. doi: 10.3390/ijms23126789 PMC9223531 35743233

[B22] Guttman J. A. Finlay B. B. (2009). Tight junctions as targets of infectious agents. Biochim. Biophys. Acta (BBA) - Biomembranes 1788, 832–841. doi: 10.1016/j.bbamem.2008.10.028 19059200

[B23] Haberkorn B. Fromm M. F. König J. (2021). Transport of drugs and endogenous compounds mediated by human OCT1: studies in single- and double-transfected cell models. Front. Pharmacol. 12. doi: 10.3389/fphar.2021.662535 PMC8100673 33967805

[B24] Han X. Sun J. Wang Y. He Z. (2015). PepT1, ASBT-linked prodrug strategy to improve oral bioavailability and tissue targeting distribution. Curr. Drug Metab. 16, 71–83. doi: 10.2174/1389200216666150401110754 25828592

[B25] Hayes B. J. Daetwyler H. D. (2019). 1000 bull genomes project to map simple and complex genetic traits in cattle: applications and outcomes. Annu. Rev. Anim. Biosci. 7, 89–102. doi: 10.1146/annurev-animal-020518-115024 30508490

[B26] Heitzmann D. Warth R. (2008). Physiology and pathophysiology of potassium channels in gastrointestinal epithelia. Physiol. Rev. 88, 1119–1182. doi: 10.1152/physrev.00020.2007 18626068

[B27] Helmy Y. A. Hafez H. M. (2022). Cryptosporidiosis: from prevention to treatment, a narrative review. Microorganisms 10. doi: 10.3390/microorganisms10122456 PMC9782356 36557709

[B28] Hodges K. Gill R. (2010). Infectious diarrhea. Gut Microbes 1, 4–21. doi: 10.4161/gmic.1.1.11036 21327112 PMC3035144

[B29] Johnson D. Bayele H. Johnston K. Tennant J. Srai S. K. Sharp P. (2004). Tumour necrosis factor alpha regulates iron transport and transporter expression in human intestinal epithelial cells. FEBS Lett. 573, 195–201. doi: 10.1016/j.febslet.2004.07.081 15327997

[B30] Jridi I. Canté-Barrett K. Pike-Overzet K. Staal F. J. T. (2020). Inflammation and wnt signaling: target for immunomodulatory therapy? Front. Cell Dev. Biol. 8. doi: 10.3389/fcell.2020.615131 PMC7890028 33614624

[B31] Jung D. Fantin A. C. Scheurer U. Fried M. Kullak-Ublick G. A. (2004). Human ileal bile acid transporter gene ASBT (SLC10A2) is transactivated by the glucocorticoid receptor. Gut 53, 78–84. doi: 10.1136/gut.53.1.78 14684580 PMC1773940

[B32] Kayaaltı Z. Akyüzlü D. K. Söylemezoğlu T. (2015). Evaluation of the effect of divalent metal transporter 1 gene polymorphism on blood iron, lead and cadmium levels. Environ. Res. 137, 8–13. doi: 10.1016/j.envres.2014.11.008 25483413

[B33] Klaassen C. D. Aleksunes L. M. (2010). Xenobiotic, bile acid, and cholesterol transporters: function and regulation. Pharmacol. Rev. 62, 1–96. doi: 10.1124/pr.109.002014 20103563 PMC2835398

[B34] Ko E.-A. Jin B.-J. Namkung W. Ma T. Thiagarajah J. R. Verkman A. (2014). Chloride channel inhibition by a red wine extract and a synthetic small molecule prevents rotaviral secretory diarrhoea in neonatal mice. Gut 63, 1120–1129. doi: 10.1136/gutjnl-2013-305663 24052273 PMC4048772

[B35] Koller S. Kendler J. Karacs J. Wolf A. Kreuzinger C. Von Der Decken I.. (2022). SLCO4A1 expression is associated with activated inflammatory pathways in high-grade serous ovarian cancer. Front. Pharmacol. 13. doi: 10.3389/fphar.2022.946348 PMC9465617 36105223

[B36] Kong D. H. Kim Y. K. Kim M. R. Jang J. H. Lee S. (2018). Emerging roles of vascular cell adhesion molecule-1 (VCAM-1) in immunological disorders and cancer. Int. J. Mol. Sci. 19. doi: 10.3390/ijms19041057 PMC5979609 29614819

[B37] Kumar A. Chatterjee I. Anbazhagan A. N. Jayawardena D. Priyamvada S. Alrefai W. A.. (2018). Cryptosporidium parvum disrupts intestinal epithelial barrier function via altering expression of key tight junction and adherens junction proteins. Cell Microbiol. 20, e12830. doi: 10.1111/cmi.12830 29444370 PMC5980709

[B38] Laftah A. H. Sharma N. Brookes M. J. Mckie A. T. Simpson R. J. Iqbal T. H.. (2006). Tumour necrosis factor alpha causes hypoferraemia and reduced intestinal iron absorption in mice. Biochem. J. 397, 61–67. doi: 10.1042/bj20060215 16566752 PMC1479761

[B39] Lamisere H. Bhalchandra S. Kane Anne V. Zeng X.-L. Mo D. Adams W.. (2022). Differential Response to the Course of Cryptosporidium parvum Infection and Its Impact on Epithelial Integrity in Differentiated versus Undifferentiated Human Intestinal Enteroids. Infection Immun. 90, e00397–e00322. doi: 10.1128/iai.00397-22 PMC9671013 36286526

[B40] Lin J.-T. Hsiao K.-J. Chen C.-Y. Wu C.-C. Lin S.-J. Chou Y.-Y.. (2011). High resolution melting analysis for the detection of SLC25A13 gene mutations in Taiwan. Clinica Chimica Acta 412, 460–465. doi: 10.1016/j.cca.2010.11.027 21134364

[B41] Lin L. Yee S. W. Kim R. B. Giacomini K. M. (2015). SLC transporters as therapeutic targets: emerging opportunities. Nat. Rev. Drug Discovery 14, 543–560. doi: 10.1038/nrd4626 26111766 PMC4698371

[B42] Lombardelli J. A. Tomazic M. L. Schnittger L. Tiranti K. I. (2019). Prevalence of Cryptosporidium parvum in dairy calves and GP60 subtyping of diarrheic calves in central Argentina. Parasitol. Res. 118, 2079–2086. doi: 10.1007/s00436-019-06366-y 31187226 PMC7087732

[B43] Love M. I. Huber W. Anders S. (2014). Moderated estimation of fold change and dispersion for RNA-seq data with DESeq2. Genome Biol. 15, 550. doi: 10.1186/s13059-014-0550-8 25516281 PMC4302049

[B44] McCole D. F. (2014). IBD candidate genes and intestinal barrier regulation. Inflammation Bowel Dis. 20, 1829–1849. doi: 10.1097/mib.0000000000000090 PMC4357271 25215613

[B45] Mercado-Lubo R. McCormick B. A. (2010). The interaction of gut microbes with host ABC transporters. Gut Microbes 1, 301–306. doi: 10.4161/gmic.1.5.12925 21327038 PMC3023614

[B46] Misselwitz B. Butter M. Verbeke K. Fox M. R. (2019). Update on lactose malabsorption and intolerance: pathogenesis, diagnosis and clinical management. Gut 68, 2080–2091. doi: 10.1136/gutjnl-2019-318404 31427404 PMC6839734

[B47] Mrsny R. J. Gewirtz A. T. Siccardi D. Savidge T. Hurley B. P. Madara J. L.. (2004). Identification of hepoxilin A3 in inflammatory events: a required role in neutrophil migration across intestinal epithelia. Proc. Natl. Acad. Sci. U.S.A. 101, 7421–7426. doi: 10.1073/pnas.0400832101 15123795 PMC409934

[B48] Nies A. T. Keppler D. (2007). The apical conjugate efflux pump ABCC2 (MRP2). Pflügers Archiv - Eur. J. Physiol. 453, 643–659. doi: 10.1007/s00424-006-0109-y 16847695

[B49] Pazos M. Siccardi D. Mumy K. L. Bien J. D. Louie S. Shi H. N.. (2008). Multidrug resistance-associated transporter 2 regulates mucosal inflammation by facilitating the synthesis of hepoxilin A3. J. Immunol. 181, 8044–8052. doi: 10.4049/jimmunol.181.11.8044 19017997 PMC2596662

[B50] Plecko B. Steinfeld R. (2017). “46 - disorders of vitamin metabolism,” in Swaiman’s pediatric neurology, Sixth Edition. Eds. Swaiman K. F. Ashwal S. Ferriero D. M. Schor N. F. Finkel R. S. Gropman A. L. Pearl P. L. Shevell M. I. (Amsterdam, The Netherlands: Elsevier), 373–382. doi: 10.1016/B978-0-323-37101-8.00046-1

[B51] Rios-Arce N. D. Collins F. L. Schepper J. D. Steury M. D. Raehtz S. Mallin H.. (2017). Epithelial barrier function in gut-bone signaling. Adv. Exp. Med. Biol. 1033, 151–183. doi: 10.1007/978-3-319-66653-2_8 29101655 PMC5742533

[B52] Schnell U. Cirulli V. Giepmans B. (2013). EpCAM: Structure and function in health and disease. Biochim. Biophys. Acta 1828. doi: 10.1016/j.bbamem.2013.04.018 23618806

[B53] Schumann T. König J. Henke C. Willmes D. M. Bornstein S. R. Jordan J.. (2020). Solute carrier transporters as potential targets for the treatment of metabolic disease. Pharmacol. Rev. 72, 343–379. doi: 10.1124/pr.118.015735 31882442

[B54] Sheng L. Luo Q. Chen L. (2022). Amino acid solute carrier transporters in inflammation and autoimmunity. Drug Metab. Disposition 50, 1228–1237. doi: 10.1124/dmd.121.000705 35152203

[B55] Sun M. Wu W. Liu Z. Cong Y. (2017). Microbiota metabolite short chain fatty acids, GPCR, and inflammatory bowel diseases. J. Gastroenterol. 52, 1–8. doi: 10.1007/s00535-016-1242-9 27448578 PMC5215992

[B56] Thiagarajah J. R. Donowitz M. Verkman A. S. (2015). Secretory diarrhoea: mechanisms and emerging therapies. Nat. Rev. Gastroenterol. Hepatol. 12, 446–457. doi: 10.1038/nrgastro.2015.111 26122478 PMC4786374

[B57] Tomita Y. Dorward H. Yool A. J. Smith E. Townsend A. R. Price T. J.. (2017). Role of aquaporin 1 signalling in cancer development and progression. Int. J. Mol. Sci. 18. doi: 10.3390/ijms18020299 PMC5343835 28146084

[B58] Tsukita S. Furuse M. Itoh M. (2001). Multifunctional strands in tight junctions. Nat. Rev. Mol. Cell Biol. 2, 285–293. doi: 10.1038/35067088 11283726

[B59] Urrutia P. Aguirre P. Esparza A. Tapia V. Mena N. P. Arredondo M.. (2013). Inflammation alters the expression of DMT1, FPN1 and hepcidin, and it causes iron accumulation in central nervous system cells. J. Neurochem. 126, 541–549. doi: 10.1111/jnc.12244 23506423

[B60] Usuda H. Okamoto T. Wada K. (2021). Leaky gut: effect of dietary fiber and fats on microbiome and intestinal barrier. Int. J. Mol. Sci. 22. doi: 10.3390/ijms22147613 PMC8305009 34299233

[B61] Vanakker O. Hosen M. De Paepe A. (2013). The ABCC6 transporter: what lessons can be learnt from other ATP-binding cassette transporters? Front. Genet. 4. doi: 10.3389/fgene.2013.00203 PMC3797522 24137173

[B62] Veshkini A. Dengler F. Bachmann L. Liermann W. Helm C. Ulrich R.. (2024). Cryptosporidium parvum infection alters the intestinal mucosa transcriptome in neonatal calves: implications for immune function. Front. Immunol. 15. doi: 10.3389/fimmu.2024.1351427 PMC10839036 38318169

[B63] Yang N. Dong Y.-Q. Jia G.-X. Fan S.-M. Li S.-Z. Yang S.-S.. (2020). ASBT(SLC10A2): A promising target for treatment of diseases and drug discovery. Biomedicine Pharmacotherapy 132, 110835. doi: 10.1016/j.biopha.2020.110835 33035828

[B64] Yap A. S. Brieher W. M. Gumbiner B. M. (1997). Molecular and functional analysis of cadherin-based adherens junctions. Annu. Rev. Cell Dev. Biol. 13, 119–146. doi: 10.1146/annurev.cellbio.13.1.119 9442870

[B65] Zhang J. Li S. Deng F. Baikeli B. Yu W. Liu G. (2019). Distribution of aquaporins and sodium transporters in the gastrointestinal tract of a desert hare, Lepus yarkandensis. Sci. Rep. 9, 16639. doi: 10.1038/s41598-019-53291-2 31719660 PMC6851143

[B66] Zheng M. Sun S. Zhou J. Liu M. (2021). Virulence factors impair epithelial junctions during bacterial infection. J. Clin. Lab. Anal. 35, e23627. doi: 10.1002/jcla.23627 33070380 PMC7891540

[B67] Zhou J. Xie Z. Cui P. Su Q. Zhang Y. Luo L.. (2020). SLC1A1, SLC16A9, and CNTN3 are potential biomarkers for the occurrence of colorectal cancer. BioMed. Res. Int. 2020, 1204605. doi: 10.1155/2020/1204605 32566650 PMC7273407

[B68] Zhu C. Chen Z. Jiang Z. (2016). Expression, distribution and role of aquaporin water channels in human and animal stomach and intestines. Int. J. Mol. Sci. 17. doi: 10.3390/ijms17091399 PMC5037679 27589719

[B69] Zierfuss B. Buda A. Villoria-González A. Logist M. Fabjan J. Parzer P.. (2022). Saturated very long-chain fatty acids regulate macrophage plasticity and invasiveness. J. Neuroinflamm. 19, 305. doi: 10.1186/s12974-022-02664-y PMC9759912 36528616

