# Supplementary material for: Cryptosporidium parvum infection alters the intestinal mucosa transcriptome in neonatal calves: impacts on epithelial barriers and transcellular transport systems
Source: Front Cell Infect Microbiol. 2024 Dec 4;14:1495309. doi: 10.3389/fcimb.2024.1495309 (PMC11656319; doi:10.3389/fcimb.2024.1495309)
Supplement: Supplementary file 7 [file Table7.docx]

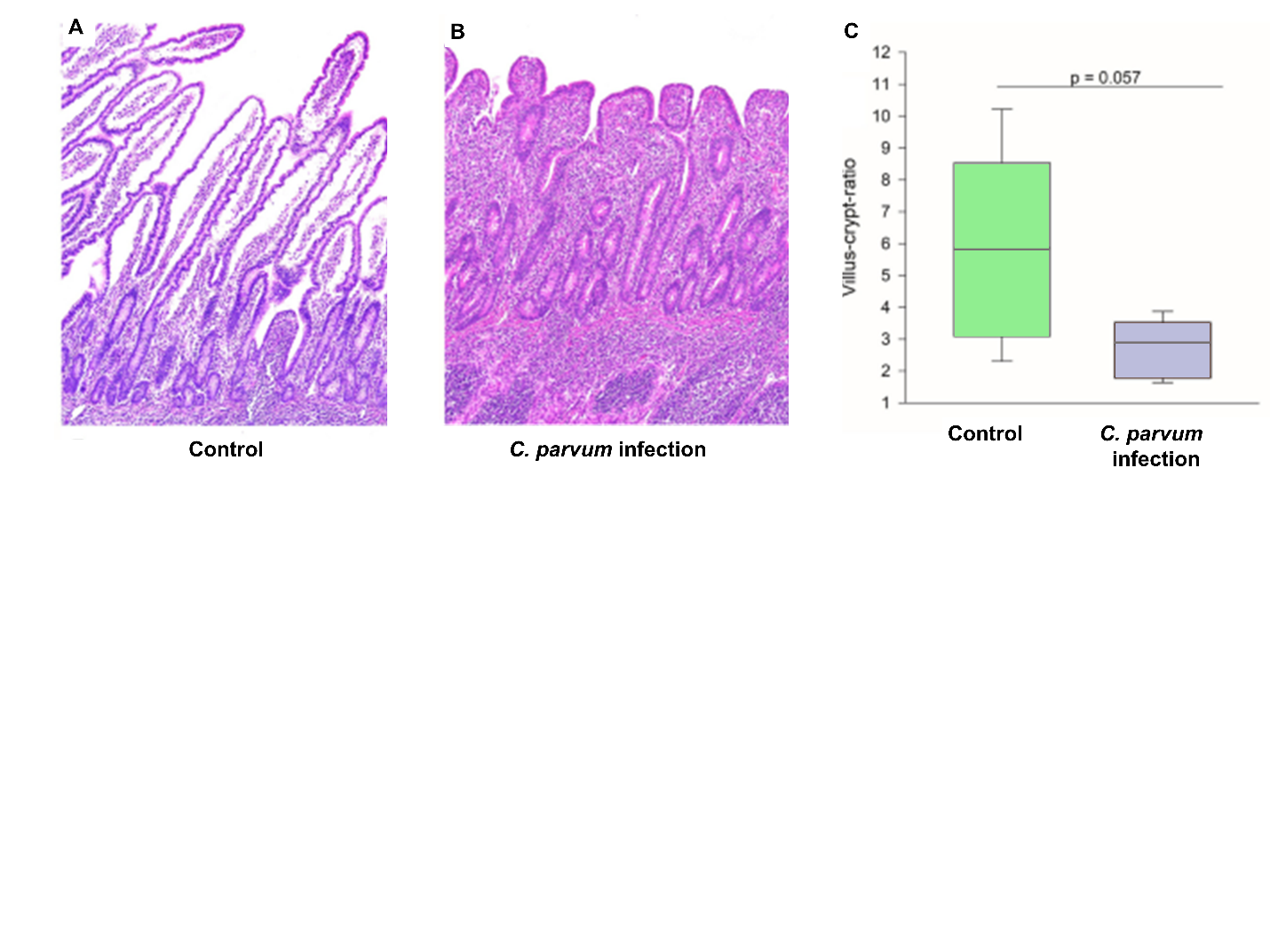


Supplementary Figure S1. This figure is adopted from our previous publication (Dengler et al., 2023). We found that the villus length of *C. parvum*-infected (B) calves was markedly reduced compared to control calves (A); scale bar = 200 mm, hematoxylin-eosin staining. (C) At macroscopic enteritis, the villus-crypt-ratio in the jejunum of infected calves is decreased compared to control calves. N = 5, Student’s t-test. Boxes show median and percentiles plus error bars.
