# Supplementary material for: Cryptosporidium parvum infection alters the intestinal mucosa transcriptome in neonatal calves: impacts on epithelial barriers and transcellular transport systems
Source: Front Cell Infect Microbiol. 2024 Dec 4;14:1495309. doi: 10.3389/fcimb.2024.1495309 (PMC11656319; doi:10.3389/fcimb.2024.1495309)
Supplement: Supplementary file 9 [file Table9.docx]

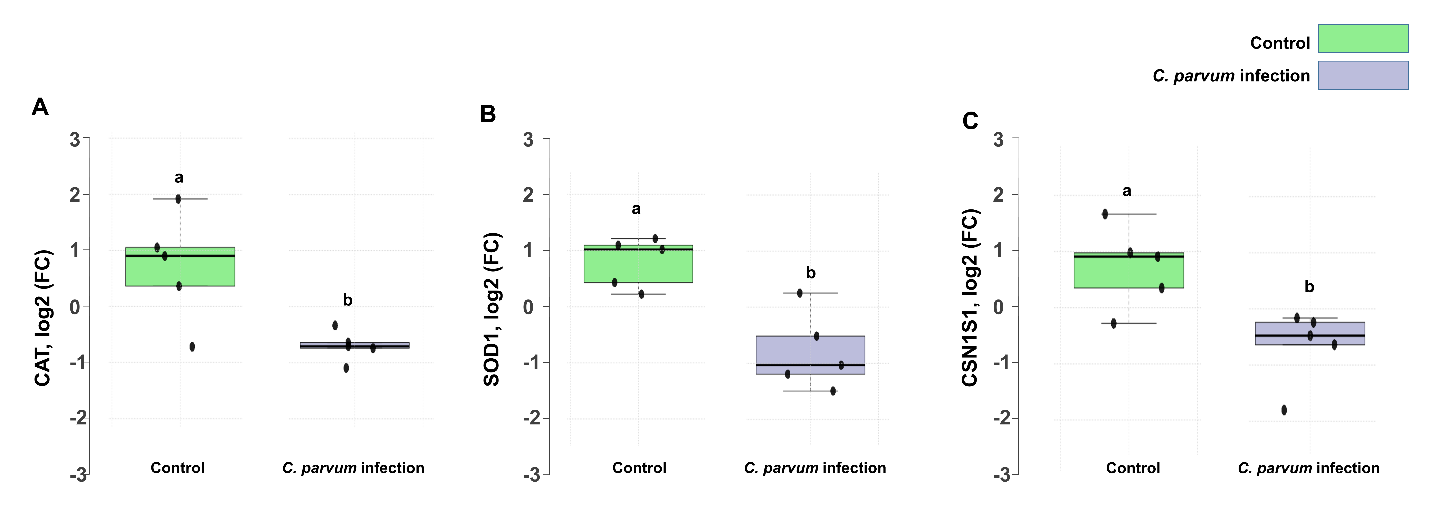


Supplementary Figure S9. Boxplots showing the comparative abundance (LC-MS/MS) of proteins associated with oxidative stress (anti-oxidants) in jejunum samples of control and infected calves. The dots represent individual claves. The black line in the box represents the mean and the error bars represent standard deviation. Different letters indicate statistically significant differences (*P* < 0.05). A, CAT (catalase), B, SOD1 (superoxide dismutase type 1), C, CSN1S1 (casein alpha S1).
