# Supplementary material for: Cryptosporidium parvum infection alters the intestinal mucosa transcriptome in neonatal calves: impacts on epithelial barriers and transcellular transport systems
Source: Front Cell Infect Microbiol. 2024 Dec 4;14:1495309. doi: 10.3389/fcimb.2024.1495309 (PMC11656319; doi:10.3389/fcimb.2024.1495309)
Supplement: Supplementary file 10 [file Table10.docx]

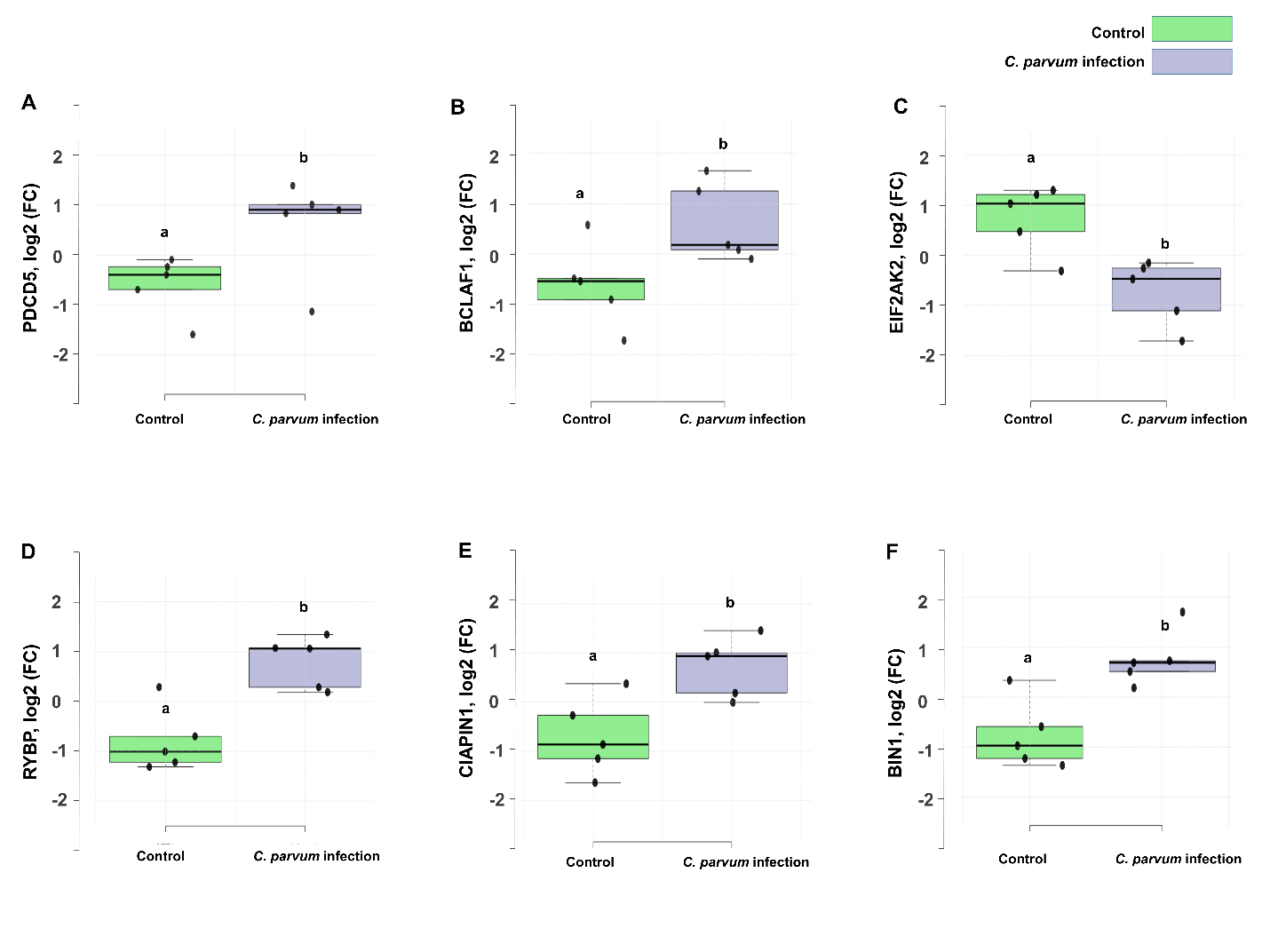


Supplementary Figure S10. Boxplots showing the comparative abundance (LC-MS/MS) of proteins associated with apoptosis and programmed cell death in the jejunum samples of control and infected calves. The dots represent individual claves. The black line in the box represents the mean and the error bars represent standard deviation. Different letters indicate statistically significant differences (*P* < 0.05). PDCD5 (Programmed cell death protein 5); BCLAF1 (BCL2 associated transcription factor 1); EIF2AK2 (Eukaryotic translation initiation factor 2-alpha kinase 2), RYBP (RING1 and YY1 binding protein); CIAPIN1 (cytokine induced apoptosis inhibitor 1); BIN1 (Bridging integrator 1).
